# Comparison of Corneal Collagen Cross-Linking and Voriconazole Treatments in Experimental Fungal Keratitis for *Aspergillus fumigatus*

**DOI:** 10.3389/fmed.2022.869429

**Published:** 2022-06-28

**Authors:** Zhennan Zhao, Xueli Chen, Yi Shao, Tingting Shao

**Affiliations:** ^1^Eye Institute and Department of Ophthalmology, Eye Ear Nose and Throat Hospital of Fudan University, Shanghai, China; ^2^NHC Key Laboratory of Myopia Fudan University, Laboratory of Myopia, Chinese Academy of Medical Sciences, Shanghai, China; ^3^Key Laboratory of Visual Impairment and Restoration of Shanghai, Shanghai, China; ^4^Department of Ophthalmology, The First Affiliated Hospital of Nanchang University, Nanchang, China

**Keywords:** corneal cross-linking (CXL), fungal keratitis, *Aspergillus fumigatus*, rabbit, voriconazole

## Abstract

**Aims:**

To compare the antifungal efficacy of corneal cross-linking (CXL) and voriconazole in experimental *Aspergillus* keratitis models.

**Methods:**

Thirty-nine New Zealand rabbits were divided into three groups: a control group, a voriconazole group (M group), and a voriconazole combined with CXL group (CXL-M group). The ulcer area was measured via slit lamp imaging, the corneal and corneal epithelial thickness, and ulcer depth was measured via anterior segment optical coherence tomography (AS-OCT). The existence time of the hyphae was observed via *in vivo* confocal microscopy (IVCM), and the cornea was taken for pathological examination after modeling and at the end of the study to determine the hyphae and corneal repair. The observation times were as follows: at successful modeling and at 1, 4, 7, 14, 21, and 28 days after intervention.

**Results:**

In the CXL-M group, ulcer area and depth decreased continuously from Day 4 to Day 28 after CXL (all *P* < 0.05). In the CXL-M group, ulcer area and depth were smaller than those in the other two groups from Day 4 to Day 21 after CXL (all *P* < 0.05, except ulcer area in the CXL-M vs. M group on Day 21). The duration of hyphae in the CXL-M group was significantly shorter than in the other two groups (*P* = 0.025). On Day 28, in CXL-M group, corneal thickness was thicker than baseline (*P* < 0.05). Meanwhile, in CXL-M group, corneal and corneal epithelial thickness were significantly thinner than in the other two groups (*P* < 0.001). The CXL-M group had no complications, such as corneal perforation, at the end of the study.

**Conclusions:**

Voriconazole combined with CXL is effective in treating *Aspergillus*-infected keratitis. Combined therapy could effectively inhibit *Aspergillus*, accelerate corneal repair, and shorten the course of the disease.

## Introduction

Fungal keratitis is a serious blinding corneal disease that accounts for about 50% of all infectious keratitis and is the leading cause of infectious keratitis in the world (1–3). In addition, the current clinical lack of specific antifungal therapy makes treatment difficult (4, 5), especially in developing countries (6, 7). Corneal collagen cross-linking therapy (CXL) is a photochemotherapy that enhances corneal strength (8, 9). In recent years, researchers have applied it to the treatment of infectious corneal ulcers and achieved promising results (10–15). Marstin et.al. found that ultraviolet radiation plus riboflavin can inhibit *Streptococcus pneumoniae, Staphylococcus aureus, Pseudomonas aeruginosa, methicillin-resistant Staphylococcus epistasis*, and other bacteria *in vitro* (16). Bilgihan et.al. had further confirmed that corneal cross-linking has inhibitory effects on common fungi *in vitro*, such as *Fusarium, Candida* and *Aspergillus* (17). Our previous results show that CXL combined with voriconazole and natamycin has a better effect on fungal corneal ulcers than drugs alone in patients (18). However, similar to the results of other clinical observation studies (11–13, 19, 20), the therapeutic effect of CXL on a single fungus has not been evaluated.

The common infections of fungal keratitis are *Candida, Fusarium*, and *Aspergillus* (21, 22), with the latter two being more common in developing countries (23). Preliminary studies have focused on the effects of CXL on *Fusarium* and *Candida* (10–16), while studies on *Aspergillus*, which has the same high incidence rate (23, 24), are rare.

Therefore, this study constructed an animal model of *Aspergillus* infection to demonstrate whether CXL combined with voriconazole is better than voriconazole alone in the treatment of fungal keratitis caused by *Aspergillus* infection based on ulcer area, ulcer depth, hyphae number change, and pathological results.

## Materials and Methods

### Aspergillus fumigatus

*Aspergillus fumigatus* was provided by the Department of Infectious Diseases, Huashan Hospital, Affiliated with Fudan University; transplanted on Potato dextrose agar (PDA) medium; and incubated in a fungal incubator at 25°C for 5 days. After amplification, it was diluted with a small amount of saline to prepare a suspension, adjusting the concentration to that required (10^6^CFU/mL) with a turbidimetric meter.

### Laboratory Animal

Thirty-nine male New Zealand white rabbits weighing 2.5~3.0 Kg that were healthy and SPF (Special pathogen free, SPF) were selected. These rabbits were provided by the Department of Animal Medicine, School of Medicine, Fudan University (Shanghai, China). All experimental methods involved in this research follow the Declaration of Helsinki, and the animal experiments were in accordance with the Association of Vision and Ophthalmology (ARVO) regulations on the use of experimental animals in ophthalmic research and approved by the Animal Ethics Committee of Fudan University School of Medicine (Shanghai, China).

### Rabbit Corneal *Aspergillus fumigatus* Infection Model

All rabbits were given an intramuscular injection of 35 mg/kg of intramuscular ketamine hydrochloride and 5 mg/kg of xylazine for general anesthesia, and oxybucaine hydrochloride eye drops were used for local anesthesia (Santen Pharmaceutical Co., Ltd.). Then, 50 μL of 10^6^ CFU/mL *Aspergillus fumigatus* suspension were drawn with a microsyringe to inject 1/3~2/3 of the full corneal thickness of the corneal stroma. Seventy-two hours after injection, typical ulcer formation and *in vivo* confocal microscopy (IVCM; HRT3-RCM, Heidelberg Engineering, GmbH, Heidelberg, Germany) showed hyphae to be determined as *Aspergillus fumigatus* infection. This was recorded as Day 0, and experimental observations began.

### Grouping and Follow-Up

The rabbits were randomly divided into three groups: a control group (C; no antifungal treatment), a medication group (M; 1% voriconazole (Vfend IV, Pfizer Pharmaceuticals, New York, USA), 3 h/time, 4 times/day), and a medication combined with cross-linking group (CXL-M; cross-linking, followed by 1% voriconazole, 3 h/time, 4 times/day). During the study, data were collected on Days 0, 1, 4, 7, 14, 21, and 28; in addition, before modeling, all rabbits underwent anterior segment optical coherence tomography (AS-OCT; RTVue Version 6.9 Optovue Inc., Fremont, CA, USA) scans to record the corneal and corneal epithelial thickness. After successful modeling, one rabbit was executed in each group, and the remainder of the rabbits in each group were executed at the end of the study. The corneal tissue of the eye was taken for pathological analysis.

### Corneal CXL Procedure

In the CXL group, on Day 0, the corneal epithelium and necrotic tissue in the central area (diameter 9 mm, including the ulcer area) were removed with a round blunt scalpel, and 0.1% riboflavin drops (Medio-Cross riboflavin/dextran solution) were applied to the eyes for 3 min/time for 30 min. Phoenix UV-A system (Peschke Meditrade GmbH, Huenenberg, Switzerland) was applied to CXL. The ultraviolet light irradiation parameters were set to 3 mW/cm^2^, and the cross-linking time was 30 min (total UVA energy 5.4 J/cm^2^). The positioning cross was irradiated in the center of the ulcer, the diameter of the spot was adjusted to 11 mm, and 0.1% Riboflavin was added every 5 min. After the cross-linking, clindamycin was used to prevent bacterial infections.

### Ophthalmologic Examinations

A uniform magnification and scale plate was applied for slit lamp imaging, and ImageJ software was used to analyze the corneal ulcer area. The corneal cross-section images were obtained by AS-OCT. Cross-sectional images of the cornea at six angles, 0–180°, 30–210°, 60–240°, 90–270°, 120–300°, and 150–330°, were captured for each eye. The acquired data were processed by AS-OCT software to measure the following parameters: maximum ulcer depth and corneal and epithelial thickness after ulcer healing. *In vivo* confocal microscopy was used to observe the hyphae and spores of *Aspergillus fumigatus* and ulcer development in each group of corneal ulcer areas and their surroundings.

### Hematoxylin-Eosin (HE) Staining

After successful modeling and at the end of the study, corneal materials were taken, and the corneal tissue was fixed with 4% neutral formaldehyde solution, dehydrated, paraffin-embedded, and continuously sliced. The slice thickness was 3 to 4 μm. Bake slices at 60°C for 1 h, HE staining, xylene transparent, neutral gum sealing. The imageJ software was used to analyze the inflammatory cell density.

### Statistical Analysis

The Statistical Package for the Social Sciences Version 23.0 was used to analyze the data. The comparison of ulcer area and depth, corneal and corneal epithelium thickness, and inflammatory cell density between groups was performed using a multivariate analysis of a one-way analysis of variance (ANOVA), which was tested for least significant difference (LSD) if the variance was homogeneous, and Dunnett's T3 test if the variance was uneven. The area and depth of the ulcers in the groups were compared using a repeated measures ANOVA, followed by Bonferroni analysis. The comparison of corneal and corneal epithelium thickness at baseline and after 28 days of treatment in each group was performed using *t*-test. Descriptive statistical values are expressed as means ± standard deviations (±SD), with *p* < 0.05 representing a significant difference.

## Results

**Figure 1:**
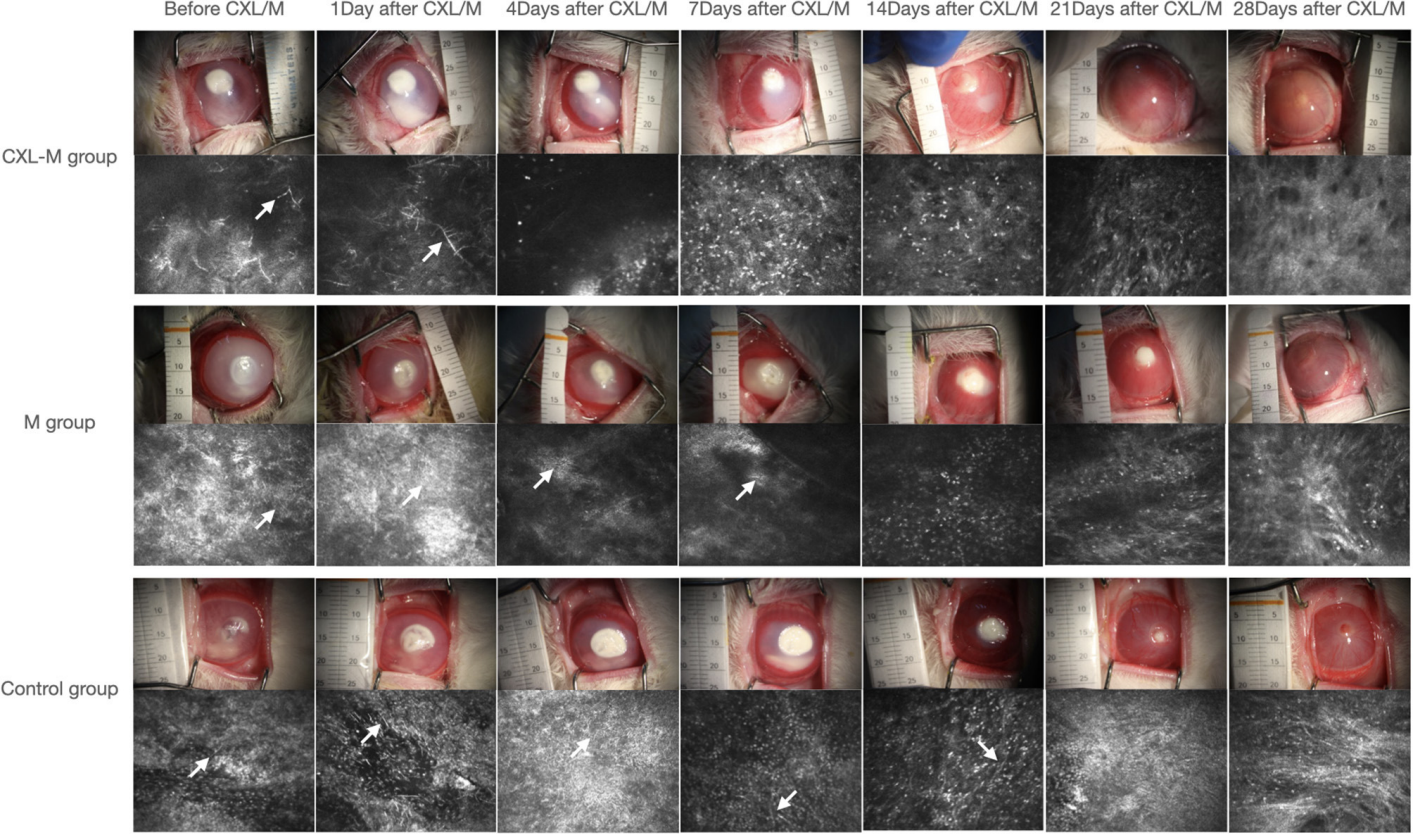
Representative images of the corneal ulcer of a representative rabbit and its corresponding IVCM in each group at all follow-up periods. The white arrow shows the fungal hypha.

**Figure 2:**
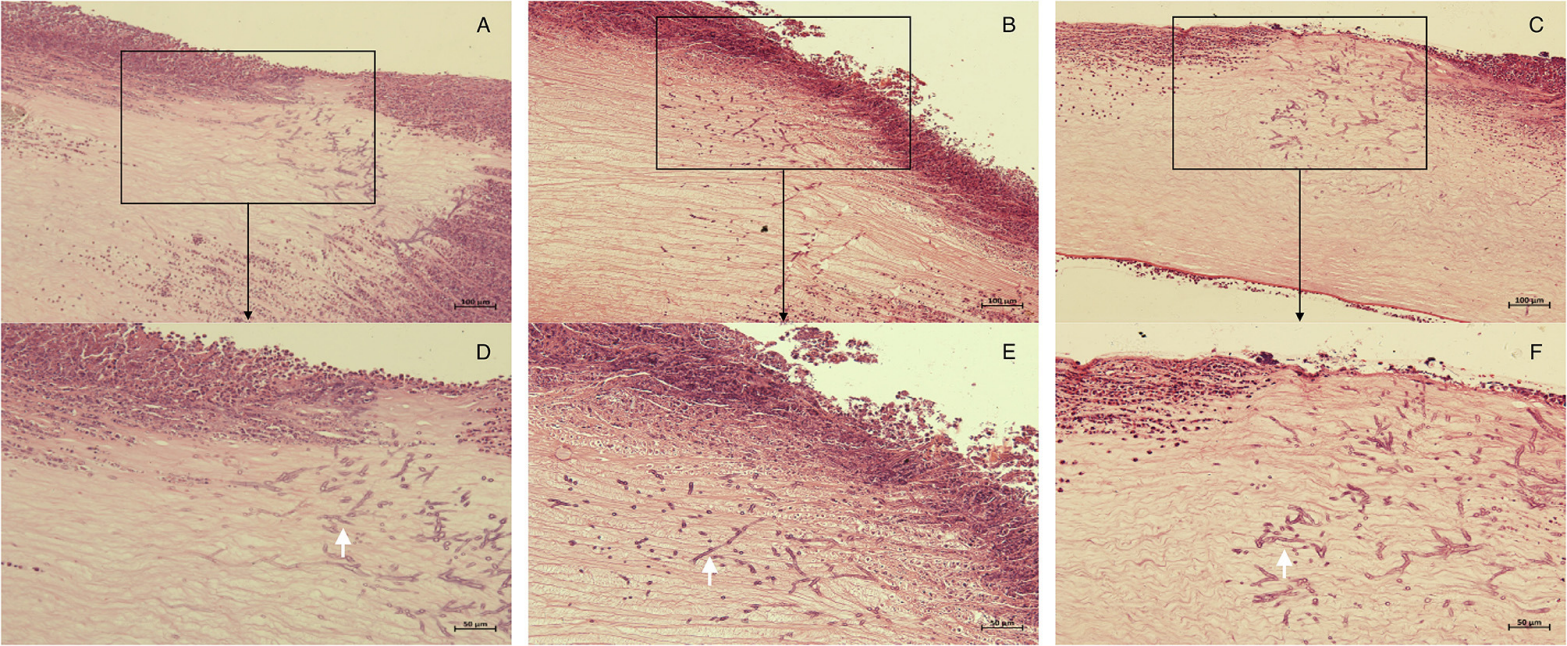
Histopathology of corneas in each group 72 h after corneal stroma injection. The corneal tissue of rabbit in each group was subject to HE staining. **(A,D)**, control group; **(B,E)**, M group; **(C,F)**, CXL-M group. **(D–F)** (X40) are the magnification of the content of the boxes in **(A–C)** (X20), respectively; and the white arrow refers to the fungal hyphae.

Seventy-two hours after corneal stroma injection, corneal epithelium defects and typical ulcer formation were seen in all three groups (Figure 1), and hyphae were seen upon IVCM (Figure 1). One corneal section was taken from each group for HE staining. Obvious hyphae can be seen (Figure 2), and was determined that the modeling of rabbit *Aspergillus fumigatus* keratitis was successful. Ultimately, twelve rabbits (twelve eyes) in each group were enrolled in the analysis. Corneal ulcer-representative images observed via slit-lamp in each group are shown in Figure 1.

**Figure 3:**
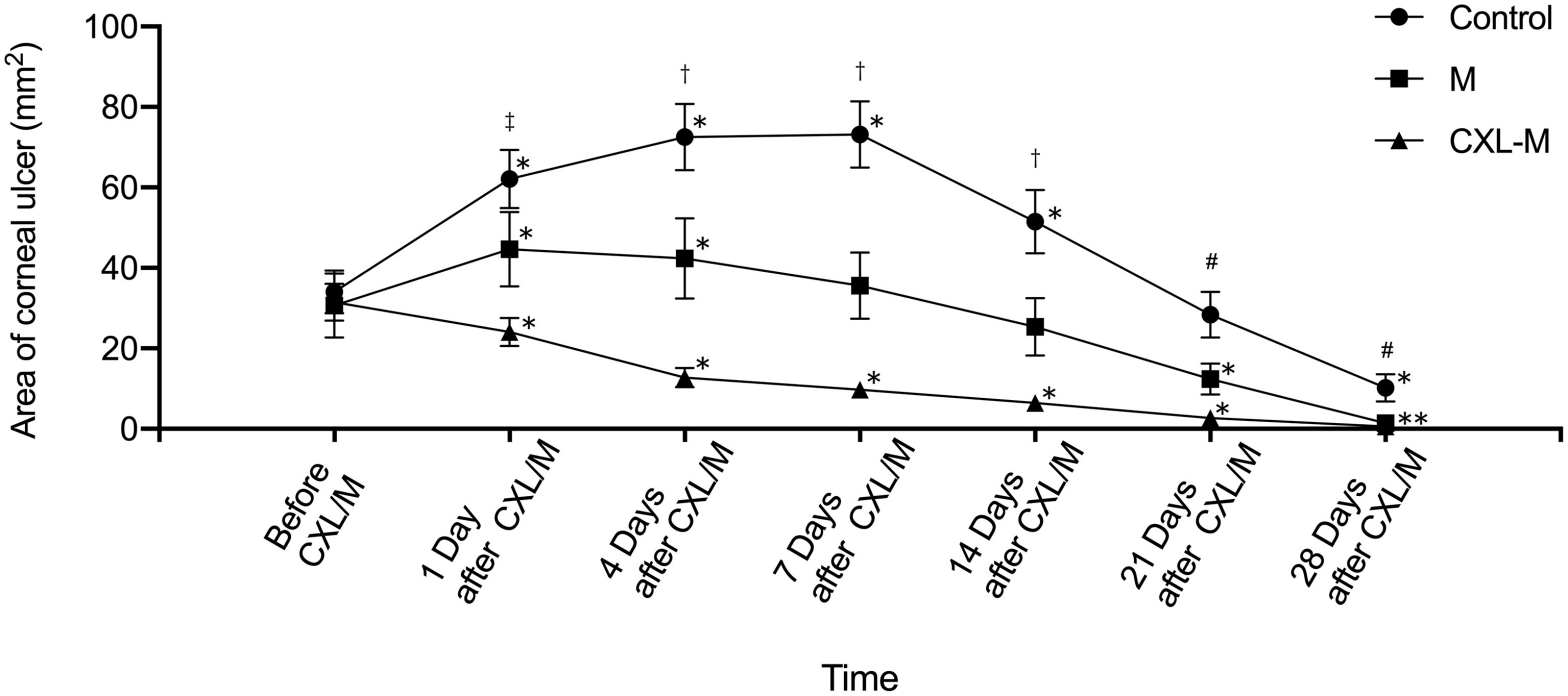
The area of corneal ulcer in three groups. *The corneal ulcer area was significantly different at different follow-up time point in each group compared with that before treatment (*P* < 0.05 for all). †There were significant differences in the areas of corneal ulcer between each group at one follow-up time point (*P* < 0.05 for all). #There were significant differences in the areas of corneal ulcer between control group and other two groups (*P* < 0.05). ^‡^There was significant difference in the area of corneal ulcer between control group and CXL-M group (*P* < 0.05).

Figure 3 shows that the ulcer area in the control group was larger than that before intervention on Days 1–14 (all *p* < 0.05), and the ulcer area reached its peak on the seventh day after intervention (73.20 ± 8.21 mm^2^). Twenty-one days after the intervention, the ulcer area was smaller than that before intervention, but this difference was not statistically significant (*P* = 0.5671). At the end of the study, the ulcer area was 10.20 ± 3.41 mm^2^, which was significantly reduced as compared with that before the intervention (*P* = 0.0005). In the M group, the ulcer area was larger than that before the medication on the first and fourth days after the medication was applied, and the difference was statistically significant (*P* = 0.0146, *P* = 0.0378). On the 7th day after the medication was provided, the ulcer area was still larger than that before the medication, but the difference was not statistically significant (*P* = 0.2311). On the 14th day after the medication was provided, the ulcer area was smaller than that before the medication was given, but the difference was not statistically significant (*P* = 0.3152); on the 21st and 28th days after the medication provided, the ulcer area was significantly smaller than before the medication (*P* = 0.0334, *P* = 0.0182). In the CXL-M group, ulcer area decreased on Day 1 after CXL, but the difference was not statistically significant (*P*-value). From postoperative Day 4 to Day 28, ulcer area was significantly smaller than that before CXL (all *P* < 0.05).

The comparison between the three groups at the same time point is shown in Figure 3. Before the intervention (including CXL and medication), the corneal ulcer area between the three groups was not statistically significant (*P* = 0.9176). On the first day after the intervention, the ulcer area in the control group (62.08 ± 7.22 mm^2^) was significantly larger than that in the CXL-M group (24.09 ± 3.52 mm^2^) (*P* = 0.0026). Additionally, in terms of ulcer area, the control group > M group and M group > CXL group, but the differences were not statistically significant (*P* = 0.2102, *P* = 0.1338, respectively). On the 4, 7, and 14th days after the intervention, the control group > M group > CXL group (all *P* < 0.05). On the 21st day after intervention, the ulcer area of control group was larger than that of M group (*P* = 0.0336) and CXL-M group (*P* = 0.0007), while the ulcer area of the M group was larger than that of the CXL group, but the difference was not statistically significant (*P* = 0.271). On the 28th day after the intervention, the difference among the three groups was the same as that on the 21st day.

**Figure 4:**
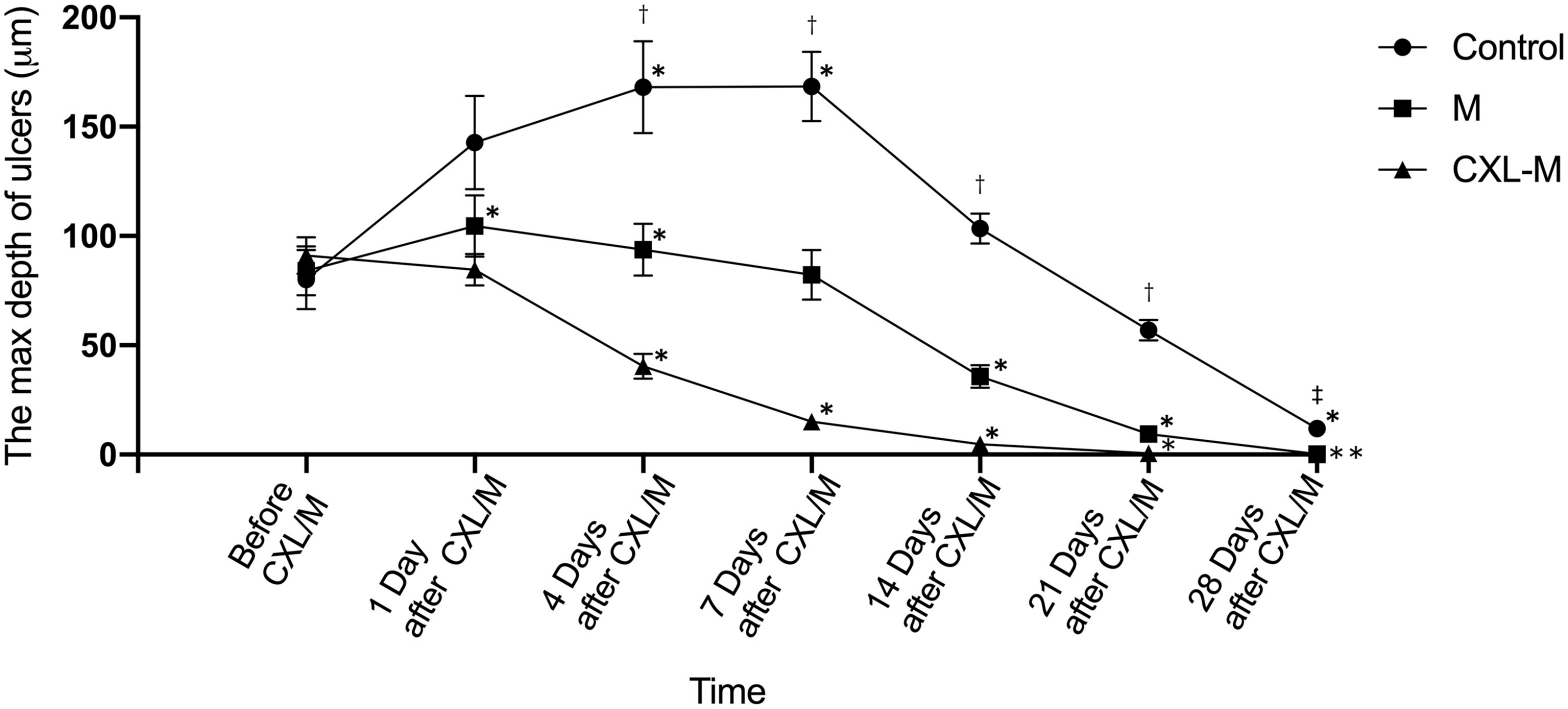
The depth of corneal ulcer in three groups. *The corneal ulcer depth was significantly different at different follow-up time point in each group compared with that before treatment (*P* < 0.05 for all). †There were significant differences in the depth of corneal ulcer between each group at one follow-up time point (*P* < 0.05 for all). ^‡^There were significant differences in the depth of corneal ulcer between control group and other two groups (*P* < 0.05).

Changes in the depth of the corneal ulcer are shown in Figure 4. In the control group, although the ulcer depth deepened significantly on the first day after the intervention, the difference was not statistically significant (*P* = 0.1208). On the 7th day after the intervention, the ulcer depth reached the peak at 168.40 ± 47.48 μm (*P* = 0.0147). On the 14th day after the intervention, the ulcer depth was deeper than that before the intervention, while on the 21st day, the ulcer depth was shallower than that before the intervention, but these differences were not statistically significant (*P* = 0.339, *P* = 0.3949, respectively). Some ulcers remained unhealed 28 days after the intervention (*n* = 2) in the control group. In the M group, the ulcers deepened on the 1st and 4th days after medication (*P* = 0.047, *P* = 0.0346); On the 7th day after medication, the ulcer depth was shallower than before medication, but this difference was not statistically significant (*P* = 0.9962). From the 14th day to the 21st day after the medication was provided, the ulcer was obviously shallower than before medication (*P* = 0.0095, *P* < 0.0001, respectively). On the 28th day, most ulcer depths were 0 (n=10). In the CXL-M group, on the first day after CXL, ulcer depth was slightly shallower than before CXL, but this difference was not statistically significant (*P* = 0.719). From Day 4 to Day 28 after CXL, ulcer depth was significantly shallower than that before CXL (all *P* < 0.05). On the 21st day after CXL, only two eyes still had mild ulcers, and at the end of the study, all the ulcers had healed.

There was no significant difference in ulcer depth between the three groups before and on the first day after the intervention (*P* = 0.8184, *P* = 0.0604, respectively). From 4 to 21 days after the intervention, there was a difference in ulcer depth between the three groups: CXL group < M group < control group (All *P* < 0.05), and this difference peaked on Day 7 (CXL group vs. M group, T = 67.33 μm, *P* = 0.0014; M group vs. control group, T = 86.11 μm, *P* < 0.0001). On the 28th day after intervention, control group > M group/CXL-M group (*P* = 0.0007, *P* < 0.0001).

Two rabbits in the control group experienced corneal perforation on the 7th and 14th days, respectively. Corneal perforation occurred in one rabbit in the M group on Day 7. No corneal perforation was observed in CXL-M group.

Representative ulcers observed via IVCM in each group are shown in Figure 1, and the average number of days of hyphae duration among the three groups was different: control group (15.75 ± 4.95 days) > M group (10.50 ± 3.742 days) > CXL group (5.125 ± 1.553 days) (*P* = 0.025).

Corneal and corneal epithelium thickness were measured via AS-OCT (Table 1). Before modeling (baseline level), there were no significant differences in corneal and corneal epithelial thickness between the three groups (*P* = 0.9586, *P* = 0.9886, respectively). On the 28th day after modeling and intervention, the corneal thickness in the three groups was significantly thicker than baseline (all *P* < 0.05). The thickness differences between the three groups were as follows: M group > control group > CXL group (*P* < 0.0001). Meanwhile, the corneal epithelium in the CXL-M group was slightly thicker than baseline, but this difference was not statistically significant (*P* = 0.0958), and corneal epithelium thicknesses in the control group and M group were significantly thicker than baseline (all *P* < 0.001). There were also differences in corneal epithelial thickness between the three groups, with the CXL-M group having thinner values than the other two groups (*P* < 0.0001).

**Table 1:** Corneal and corneal epithelium thickness in three groups.

		**Base line**	**28 days after M/CXL**	***P***
Corneal epithelium thickness (μm)	Control (n/n' = 12/8)	45.56 ± 2.186	74.89 ± 5.905	<0.0001
	M (n/n' = 12/10)	45.42 ± 2.234	79.42 ± 7.229	<0.0001
	CXL-M (n/n' = 12/12)	45.25 ± 2.121	47.89 ± 3.940	0.0958
	*P*	0.9596	<0.0001	
Corneal thickness (μm)	Control (n/n' = 12/8)	381.25 ± 24.42	608.80 ± 34.62	<0.0001
	M (n/n' = 12/10)	380.80 ± 21.68	736.60 ± 36.34	<0.0001
	CXL-M (n/n' = 12/12)	382.10 ± 17.54	517.40 ± 29.25	<0.0001
	*P*	0.9886	<0.0001	

*M, voriconazole only; CXL-M, cross-link combined with voriconazole; The test used for statistical analysis among groups was t-test and between groups was one-way ANOVA. n/n', number of rabbits at base line/ number of rabbits with perforation and non-healing excluded at 28 days after treatment*.

**Figure 5:**
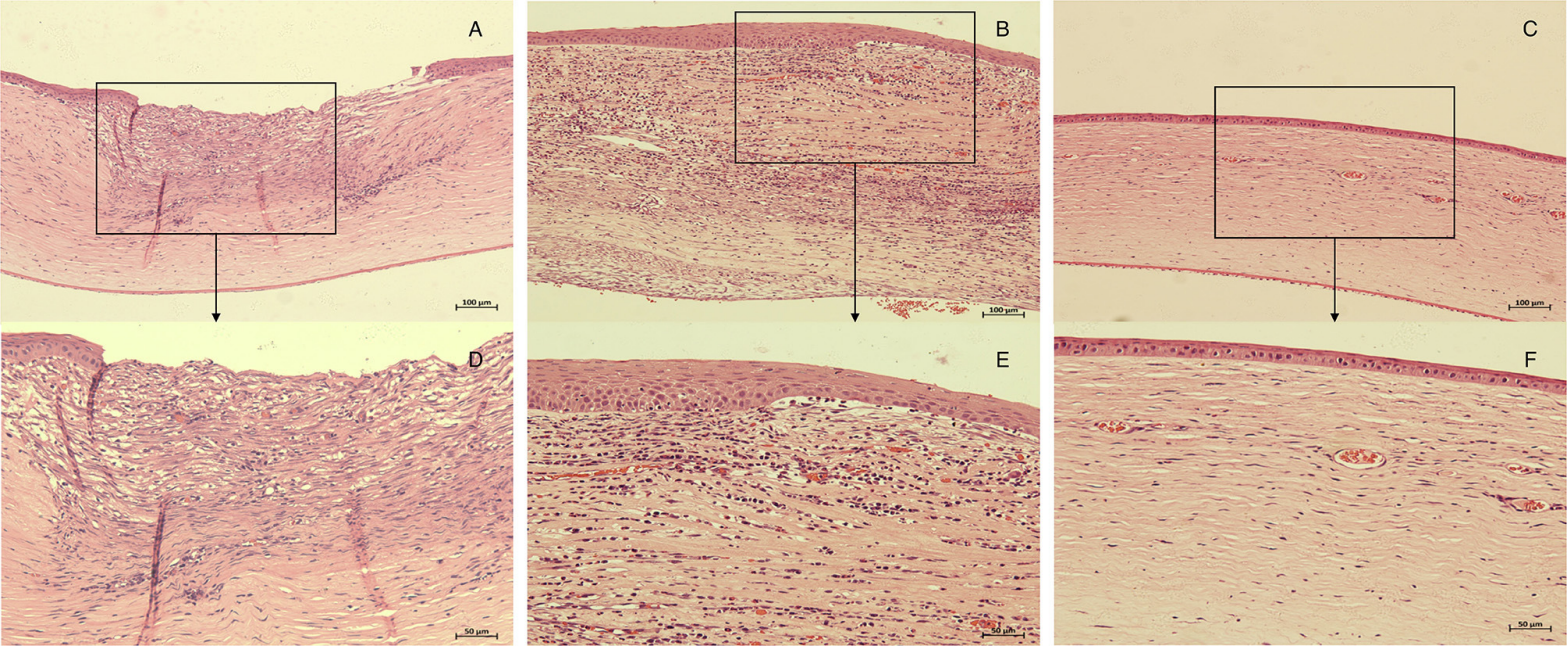
Histopathology of corneas in each group 28 days after treatment (CXL/M). The corneal tissue of rabbit in each group was subject to HE staining. **(A,D)**, control group; **(B,E)**, M group; **(C,F)**, CXL-M group. **(D–F)** (X40) are the magnification of the content of the boxes in **(A–C)** (X20), respectively.

**Figure 6:**
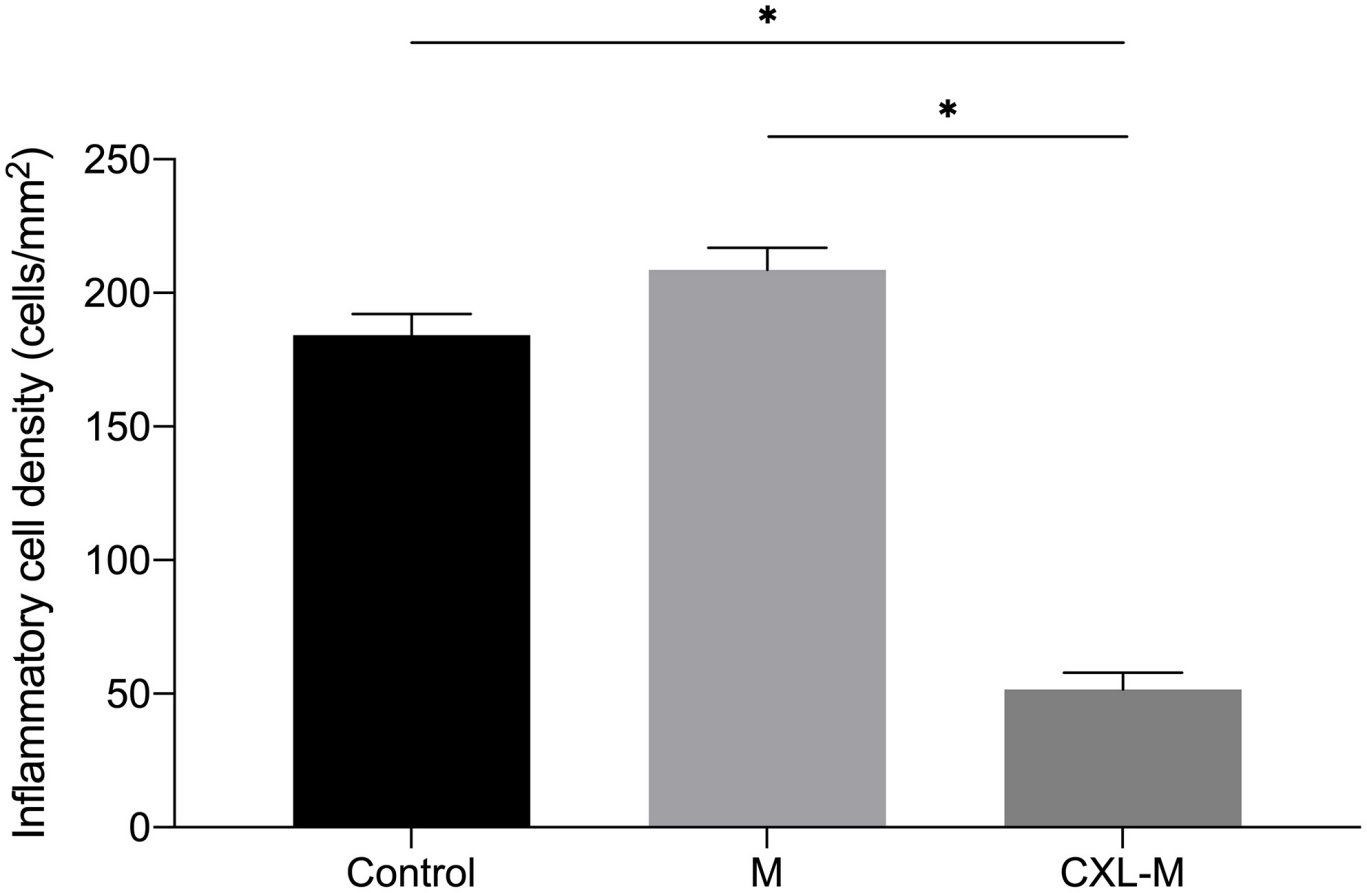
The inflammatory cell density in three groups 28 days after treatment (CXL/M). The graph shows the inflammatory cell density of the CXL-M at the end of the study was significantly less than that of other two groups (all *P* < 0.0001). *P < 0.05.

The HE-stained sections of each group at 28 days after the intervention are shown in Figure 5. In the control group, there was more than 12 corneal structural damage, scar repair, diffuse thickening of cornea, abundant proliferation of corneal stromal fibroblasts, fibrosis and extremely chaotic fiber arrangement, massive inflammatory cells and neovascularization, a thickened epithelial layer with a multi-layered cell arrangement, and local epithelial defect. In the M group, there was diffuse thickening of the entire cornea, with a large amount of inflammatory cell infiltration and neovascularization; a large number of fibroblasts in the stromal layer with chaotic fiber arrangement; and obvious thickening of the corneal epithelium with multilayer cells. In the CXL group, the proliferation of fibrous tissue in the stroma was regular, some neovascularization could be seen locally, and the corneal epithelium was flat and arranged in a single layer. The inflammatory cell density of CXL-M group was significantly lower than that of the other two groups (*P* < 0.001, Figure 6).

## Discussion

This study used conventional voriconazole combined with CXL in treating rabbit fungal keratitis infected by *Aspergillus*, which is the first drug-combined-with-CXL study for an animal model of *Aspergillus* infection. We found that voriconazole combined with CXL can significantly curb the development of corneal ulcers caused by *Aspergillus* infection, accelerate healing, and reduce complications.

In this study, the average hypha duration for the CXL-M group was significantly shorter than that of the other two groups. Bilgihan et al. and Sun et al. demonstrated that CXL has inhibitory effects on *Aspergillus, Fusarium*, and *Candida in vitro* (17, 25). During the crosslinking process, oxygen radicals produced by the photooxidation of riboflavin via ultraviolet radiation can damage the genetic material of the fungus, destroy the DNA of the pathogen, and kill the pathogen (26, 27); at the same time, the reaction process consumes oxygen, creating an oxygen-deficient environment, affecting the replication and growth of the fungus (27, 28). Interestingly, Sauer et al. found that CXL had no antifungal effect on *Candida, Fusarium*, and *Aspergillus in vitro*, while after pretreatment with amphotericin B, CXL had a significant antifungal effect (29). The author believes that cross-linking promotes the distribution of amphotericin B, which helps anti-fungi (29). However, the manner of adding riboflavin in their study may have led to the result that CXL alone had no antifungal activity *in vitro* (25). It is also believed that the results obtained via *in vitro* studies cannot effectively reflect effectiveness *in vivo* (29). Therefore, tissue culture or animal studies may better detect the effectiveness of cross-linking on infectious keratitis. In this animal study, our results show that the combination of voriconazole and CXL had excellent anti-*Aspergillus* effects, controlling the infection earlier and more effectively compared to voriconazole treatment alone.

The ulcer area in the control group and M group both increased from the first day after the intervention and continued to increase for 14 and 7 days respectively. By the end of the study, some rabbits still had incomplete ulcer healing in both groups. However, the area of ulcers in the CXL-M group began to shrink from the first day of the intervention, and all the rabbits had ulcer healed at the end of the experiment. Further analysis of the ulcer area between the three groups at the same time point showed that the ulcer area of the CXL-M group was significantly smaller than that of the other two groups during the entire study after the intervention (except for the 1st and 28th days of M group). The changes in the depth of ulcers within and between the three groups showed a trend similar to that of the area of corneal ulcers. It is suggested that voriconazole combined with CXL is significantly better than voriconazole treatment alone in terms of onset time and ulcer healing (ulcer area and depth).

There are few randomized controlled studies on CXL in the treatment of fungal corneal ulcers, and the previous results are also conflicting. Ozdemir et al. and Galperin et al. report the effectiveness of cross-linking against *Fusarium* and *Candida* infectious keratitis in rabbits (14, 30). Similarly, cross-linking was also beneficial in treating *Fusarium* infectious keratitis in mice (31). Although our previous clinical study and Jeyalatha Mani et.al both reported that CXL had a significant adjuvant effect of CXL in treating fungal keratitis compared to standalone antifungal treatment (18, 32), further analysis of various fungal species has not been conducted. Uddaraju et al. and Price et al. found that CXL has a poor response to the healing of fungal corneal ulcers (20, 33). These researchers believe that the deeper and larger ulcers before the intervention are responsible for the poor response. Vajpayee et al. observed the healing time of fungal corneal ulcers after CXL and found no difference between CXL and medication alone (34). However, in comparing the healing time of aspergillus-infected corneal ulcer in their study, the CXL group was superior to the medication group. Thus far, there has been no animal study or clinical observation report on the effects of CXL on *Aspergillus* infection alone. This study is the first drug combination cross-linking study for Aspergillus infectious corneal ulcers in rabbit; the results showed that the healing of ulcers in the CXL-M group was significantly better than that in the M group and the control group.

*Aspergillus* can produce proteases, such as metalloproteinase and serine proteinase, which can degrade collagen (35). However, the large amount of singlet oxygen produced during CXL may damage the protease and protect the corneal stroma (36). Corneal collagen cross-linking can also change the tertiary structure between collagen fibers, thereby preventing protease from acting on its specific enzymatic site (37). On the other hand, corneal collagen crosslinking can improve the mechanical strength and stability of the cornea and enhance its tolerance of enzyme digestion (36, 38). After CXL, the cornea hardens, and its permeability will decrease, which prevent the invasion of fungi into the deep cornea (17, 38, 39).

Corneal perforation was observed in both the control group and the M group during follow-up, while no corneal perforation was observed in the CXL-M group in this study. Said et al. report the effectiveness of CXL in severe infectious keratitis with kerolysis (38). In their study, no corneal perforation occurred in the cross-linking group, and about 40% of cases were aspergillus infections. In addition, both Z. Li et al. and Galperin et al. reported that CXL was effective in treating fungal keratitis without complications (13, 30). However, it was also found that the perforation rate of patients with deep stromal fungal keratitis after CXL was higher than that in the control group (33). These researchers suggested that CXL responds poorly to deeper ulcers and may even increase the risk of perforation for deeper corneal ulcers (12, 20, 33). The differences in the reported results may be caused by the various fungal species and their abilities to invade the corneal stroma. In addition, in animal studies, the initial depth of corneal ulcer may also be different after modeling, and most of the previous studies did not measure ulcer depth. The use of different cross-linking schemes may also have contributed to this difference. In addition, Uddaraju et al. believe that CXL may have specific effects on certain fungi (33).

In this study, the ulcer depth was quantitatively measured by AS-OCT, and it was found that the average ulcer depth in the CXL-M group was 91 μm before intervention, while no abnormality of the corneal endothelium was found in pathological sections of the CXL-M group. Shetty et al. suggest that the superficial corneal stroma infiltration of the anterior one-third depth responds better to CXL (12). Therefore, we speculated that voriconazole combined with CXL can safely and effectively control *aspergillus*-infected corneal ulcers at an ulcer depth of at least 90 μm in rabbit. Further studies are needed to confirm whether use for ulcers beyond this depth is still effective and safe.

On the 28th day after the intervention, although the corneal thickness of all three groups were thicker than the baseline before modeling, the CX-M group was significantly thinner than that of the other two groups. The corneal epithelial thickness of the CXL-M group was also thinner than that of the other two groups, but there was no significant difference between the CXL-M group and baseline level, while the other two groups were thicker than the baseline level. This result is similar to the results of our previous clinical observation research (18). We speculated that the corneal inflammation in the CXL-M group was milder at this time and that the corneal shape was closer to the normal. Pathological sections also confirmed that the corneal epithelium of the cross-linking group was monolayer; stromal fiber was regular; and inflammatory cells, neovascularization, and fibroblasts were significantly less than in the other two groups at this time.

The corneal epithelial cells recognize fungi through pattern recognition receptors (40), such as Toll-like receptors (TLR) and C-type Lectin receptors (CLR). *Aspergillus fumigatus* encounter during fungal keratitis have been reported to be sensed by TLR (41), which can induce production of chemokines and recruit neutrophils (are more than 90% of the infiltrating cells) (40). Jeyalatha Mani et.al found that the expression of pro-inflammatory factors (IL-1β, IL-8, and IFN-γ) and TLRs (TLR-3/4/6) were significantly downregulated after CXL treatment in patients with fungal keratitis (32), suggesting that CXL may exerts an anti-inflammatory effect via TLRs pathway. In addition, photooxidation during CXL can inactivate leukocytes and reduce and regulate inflammatory response (42), which may also participate in the effect observed. Wollensak et al. found that cross-linking can significantly reduce the edema coefficient in the cross-linking area (43), and Holopainen et al. found that the corneal thickness decreased by an average of 19 ± 7% after crosslinking (44), suggesting that the anti-edema effect of cross-linking may also be one of the reasons for thinner corneal thickness in the CXL-M group as compared with the other two groups.

The sample size of this study is small, and the follow-up time is short. The results must be further verified in subsequent large-sample and long-term studies. Currently, no report has recommended CXL as a standalone first-line therapy, so this study did not include CXL treatment alone group, and relevant studies will be carried out in the future. To date, there is an absolute empiric use and no protocol consensus and agreement or fungal-agent-specific CXL or customized CXL nomogram. This experimental setting finally studied an organism-specific response to clarify that the adjuvant effect of CXL may be dependent on the specific fungal organism. In addition, only traditional CXL (3 mW/cm^2^, total UVA energy 5.4 J/cm^2^) combined with voriconazole and voriconazole alone were compared in this study. The question of whether there are differences between different CXL protocols (such as 9 mW, 18 mW, and 30 mW CXL, etc.) and different antifungal agents (such as natamycin, amphotericin, etc.) could not be answered in this study, but subsequent studies should investigate this matter further.

This study found that voriconazole combined with corneal collagen crosslinking can significantly inhibit fungal keratitis caused by aspergillus infection and accelerate ulcer healing. It can be used as an adjunctive treatment for fungal keratitis of aspergillus infection, especially for early and superficial corneal ulcers.

## Data Availability Statement

The original contributions presented in the study are included in the article/supplementary material, further inquiries can be directed to the corresponding authors.

## Ethics Statement

The animal study was reviewed and approved by Animal Ethics Committee of Fudan University School of Medicine (Shanghai, China).

## Author Contributions

TS and YS contributed to conception and design of the study. ZZ and XC collected and measured the data. ZZ performed the statistical analysis and wrote the first draft of the manuscript. XC wrote sections of the manuscript. TS revised the manuscript. All authors contributed to manuscript revision, read, and approved the submitted version.

## Conflict of Interest

The authors declare that the research was conducted in the absence of any commercial or financial relationships that could be construed as a potential conflict of interest.

## Publisher's Note

All claims expressed in this article are solely those of the authors and do not necessarily represent those of their affiliated organizations, or those of the publisher, the editors and the reviewers. Any product that may be evaluated in this article, or claim that may be made by its manufacturer, is not guaranteed or endorsed by the publisher.
